# DNA barcoding indicates the range extension in an endemic frog *Nyctibatrachus jog*, from the Western Ghats, India

**DOI:** 10.1080/23802359.2021.1955765

**Published:** 2021-07-26

**Authors:** Priti Hebbar, Anisha Anand, Gururaja K.V

**Affiliations:** a Centre for Ecological Sciences, Indian Institute of Science, Bangalore, India; b Srishti Manipal Institute of Art, Design and Technology, Bangalore, India

**Keywords:** Amphibians, distribution range, DNA barcoding, Night frogs, river basin

## Abstract

The frogs of genus *Nyctibatrachus* from the Western Ghats are endemic, with some taxa showing a narrow distribution range. *Nyctibatrachus jog* was known only from the type locality, Jog falls from Sharavathi river basin suggesting a restricted distribution. In this study, using DNA barcoding, we studied the distribution patterns of *N. jog* by sampling multiple river basins. 16S rRNA and Cytochrome b genes were used to distinguish *N. jog* from its congeners as well as to infer intra-species relationships. The results from the 16S rRNA gene showed 99% similarity of the collected individuals with the type specimen from Jog Falls confirming the identity of *N. jog*. The results indicate that *N. jog* has wide distribution extending its range in multiple river basins in the Western Ghats, India. This study also provides the Area of Occurrence and Extent of Occurrence of *N. jog* which could help in developing strategies for its conservation.

## Introduction

DNA barcoding has been routinely used to identify species, contributing to biodiversity research (Hebert et al. 2003; Hajibabaei et al. 2007). It has also been useful to understand the geographical distributions of populations, providing insights into the patterns of geneflow within a species (Hajibabaei et al. 2007). Using DNA barcoding, many studies have identified the new extent of the species thus expanding their distribution range (Seshadri et al. 2016; Kundu et al. 2020; Lim et al. 2020).

The Western Ghats of India is known for the high diversity of amphibians with more than 90% of the species endemic to the region. There are nine families of anurans found here, of which three families, Micrixalidae Dubois et al. (2001), Ranixalidae Dubois (1987) and Nasikabatrachidae Biju and Bossuyt (2003) are endemic to the Western Ghats while family Nyctibatrachidae Blommers-Schlösser (1993) has genera *Nyctibatrachus* and *Astrobatrachus* distributed in the Western Ghats and genus *Lankanectus* endemic to Sri Lanka. Some of these taxa have narrow distribution ranges, making them of high conservation concern (Vijayakumar et al. 2019).

The genus *Nyctibatrachus* is endemic to the Western Ghats, India. There are 36 species at present (Biju et al. 2011; Gururaja et al. 2014; Garg et al. 2017; Krutha et al. 2017). The characteristics of *Nyctibatrachus* frogs include wrinkled skin, rhomboid pupils, pointed vomerine teeth, subocular glands and a notched tongue (Biju et al. 2011). The larger *Nyctibatrachus* species are associated with torrential streams whereas the smaller *Nyctibatrachus* species are associated with marshy pools. (Biju et al. 2011). A study by Van Bocxlaer et al. (2012) revealed that the species show mountain-associated clade level endemism with a narrow distribution range. The authors mentioned that torrentially adapted *Nyctibatrachus* species disperse along the river systems but may have restricted distribution range due to the east-west orientation of the river system, thus limiting their expansion from one river system to another. However, fine-scale studies understanding the distribution range for many *Nyctibatrachus* species is lacking. Knowledge of the distribution extent can be helpful for their conservation.

*Nyctibatrachus jog* Biju et al. (2011) is an endemic frog from central Western Ghats India. The species was discovered from Jog falls, Karnataka at an elevation of 600 m by Biju et al. (2011). The species is adapted to fast-flowing streams and torrents (Biju et al. 2011). The authors mentioned that *N. jog* was found only at the type locality, Jog Falls, central Western Ghats, India. However, the exact distribution range of *N. jog* is not known. In this study, we provide additional distribution records of *N. jog,* identified using DNA barcoding methods. We also provide the Extent of Occurrence (EOO) and Area of Occurrence (AOO) for *N. jog.*

## Material and methods

**Figure 1.**
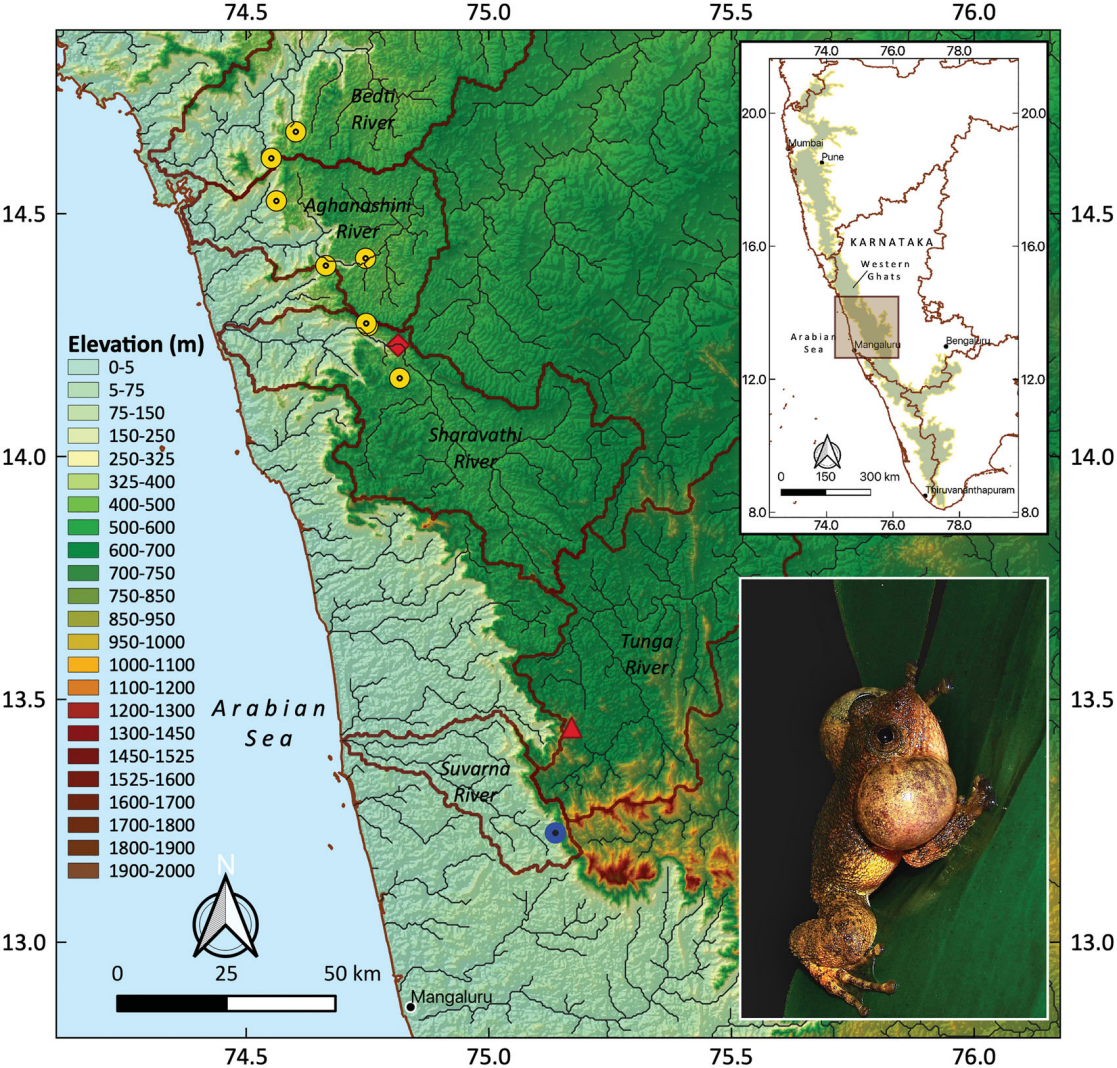
Current distribution records of *Nyctibatrachus jog* along the Western Ghats. The type locality of Jog Falls is given in rhombus. Triangle indicates the type locality as per Biju et al. (2011) latitude and longitude value, which is 150 km south of the actual type locality. Jog Falls. Yellow circles represent sites sampled in this study. Blue circle represents the presence of *N. jog* not sampled in this study.

As a part of a study on the ecology and genetics of *Nyctibatrachus* species, we sampled the streams from Bedti, Aghanashini and Sharavathi (Figure 1) river basins from the central Western Ghats of India. These rivers originate in the Western Ghats and flow westwards to join the Arabian sea. The river basins are differentiated based on their catchment areas and delineated using Digital Elevation models (DEM). 43 streams were sampled from the three river basins. The streams were sampled for both adults and tadpoles. The tadpoles were sampled using dipnets (15 cm by 15 cm). To distinguish *N. jog* from its congeners, individuals were identified by their calls, size, sex, clutch size following Biju et al. (2011), Gururaja et al. (2014) and by DNA barcoding. A small tadpole tail tip was excised and preserved in 80% ethanol for molecular work and the tadpoles were released back into the streams.

### Molecular methods

The DNA was extracted using the salt extraction method as mentioned in Vences et al. (2012). A 530 bp of 16S rRNA gene (Palumbi et al. 2002) was used for identification as it has been widely used for DNA barcoding of amphibians (Vences et al. 2005). We also used 427 bp Cytochrome b (Cyt-b) gene (mcb 398: TACCATGAGGACAAATATCATTCTG3 mcb 869: CCTCCTAGTTTGTTAGGGATTGATCG3, Verma and Singh 2002). 23 individuals were sequenced for 16S rRNA and 26 individuals were sequenced for Cyt-b. The sequences were checked for identity using nucleotide BLAST (https://blast.ncbi.nlm.nih.gov/) and deposited in GenBank (16S Accession numbers MW081889–MW081909, Cyt-b Accession numbers MW762640–MW762665). Two 16S sequences viz adult *N. jog* (Voucher number BNHS5457) with accession number JN644900 (Van Bocxlaer et al. 2012) and tadpole *N. jog (*Voucher number BNHS5900*)* with accession number KP317819 (Priti et al. 2015) were downloaded from GenBank to confirm the identity of genetic sequences generated in this study. The monophyly of *N. jog* individuals were analyzed by building phylogenetic tree for 16S rRNA gene using Bayesian inference in Mr Bayes (Ronquist and Huelsenbeck 2003) and Maximum Likelihood methods in RAxMLGUI (Silvestro and Michalak 2012). GTR + I + G was used as substitution model as selected by Partition Finder v 1.1.1 (Lanfear et al. 2012). *Lankanectes corrugatus* (Accession number AF215393) was used as an outgroup. The genetic distance was calculated using Kimura 2 parameter method in MEGA 7 (Kumar et al. 2016). To understand the distribution range of *N. jog,* the Area of Occurrence (AOO) and the Extent of Occurrence (EEO) were estimated using GeoCat (Bachman et al. 2011).

## Results

**Figure 2.**
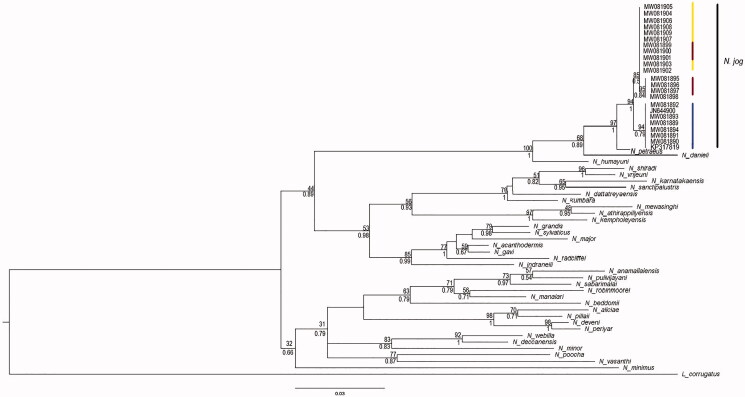
Maximum Likelihood phylogenetic tree depicting the inferred relationships among members of the genus *Nyctibatrachus* based on 530 base pairs of the mitochondrial 16S gene. The number above the branches denotes bootstrap support. The numbers below the branches denote Posterior Probability values. Blue vertical line represents Sharavathi river basin, Yellow Line represents Aghanashini river basin and Red line indicates Bedti river basin.

The new localities where *N. jog* individuals were observed are summarized in Table 1. The phylogenetic tree showed that the *N. jog* individuals formed a strong monophyly (Bootstrap support = 94, Posterior Probability = 1, Figure 2). The generated sequences showed 99.2% similarity with the published 16S rRNA sequence from the type locality, Jog falls mentioned in Biju et al. (2011), Van Bocxlaer et al. (2012) respectively (Accession number JN644900). The individuals collected from multiple localities of Sharavathi river basin clustered with the adult *N. jog* (Accession number JN644900) as well as with the tadpole N. jog (Accession number KP317819) confirming the identity of *N. jog* (Figure 2). Although the genetic data of Biju et al. (2011) matches with our study, the location mentioned as Jog falls (13.4421 N, 75.1704E) by Biju et al. (2011) falls in Tunga river basin which is 150 km away from Jog falls. This could be an error in coordinates provided by the authors. The actual co-ordinates of Jog Falls are 14.2294°N, 74.74679°E. From our study, the occurrence of the *N. jog* now extends into multiple localities in Aghanashini, Bedti and Sharavathi river basins. The maximum interspecies genetic distance was with *N. periyar* (13%) while minimum interspecific distance was with *N. petraeus* (1%). Although the interspecies distance between *N. jog* and *N. petraeus* is less for 16S r RNA gene, the genetic distance between the two species for ND1 gene was 4.5% along with the difference in morphology, based on which, they were distinguished as distinct species (Biju et al. 2011). The intraspecific genetic distance among *N. jog* individuals for 16S rRNA gene ranged from 0.00 to 0.8% (Table 2) while for the Cyt-b gene, the intraspecific genetic distance ranged between 0.00 to 1.95% (Table 3). The intraspecies genetic relationship as shown by Maximum likelihood and Bayesian methods showed populations of Sharavathi river basin as a separate clade (BS > 95, PP = 1, Figure 2) from the populations of Aghanashini and Bedti river basin whereas there was some admixture between populations of Aghanashini and Bedti river basins. Further studies understanding intra-species genetic diversity and gene flow in *N. jog* is needed. The area of occupancy (AOO) for *N. jog* is 32 km^2^ and the extent of occupancy (EOO) is 461.420 km^2^. Besides these sites, personal observations by naturalist Manu Nakathaya from Reserve Forest in Mala (Suvarna river basin) near Kudremukh also suggests the presence of *N. jog* based on morphology and call records, which is further south by 110 km from the type locality. If these points are included, the area of occupancy (AOO) for *N. jog* increases to 36 km^2^ and the extent of occupancy (EOO) increases to 1629.464 km^2^.

**Table 1.** Sampling localities of *N. jog* along with GenBank accession numbers.

						GenBank Accession numbers	
Sampling site	River basin	Latitude (°N)	Longitude (°E)	16S	Cyt-b	16S	Cyt-b
				Number of samples			
DEV	Aghanashini	14.5261	74.56192	2	5	MW081908– MW081909	MW762661– MW762665
DOD	Aghanashini	14.393	74.66388	3	5	MW081902– MW081904	MW762652– MW762656
UNCH	Aghanashini	14.4079	74.74576	3	4	MW081905–MW081907	MW762657–MW762660
KEL	Bedti	14.6685	74.60194	4	4	MW081895– MW081898	MW762647– MW762650
VAD	Bedti	14.6139	74.5515	3	1	MW081899– MW081901	MW762651
Jog Falls*	Sharavathi	13.4421	75.1704	1	–	JN644900(Van Bocxlaer et al. 2012)	–
Jog Falls	Sharavathi	14.274	74.74679	1	–	KP317819(Priti et al. 2015)	–
Jog falls	Sharavathi	14.2291	74.81263	3	–	MW081892– MW081894	–
BAR	Sharavathi	14.2693	74.75055	2	–	MW081890– MW081891	–
KATH	Sharavathi	14.2739	74.747	1	4	MW081889	MW762640–MW762643
VAT	Sharavathi	14.161	74.81609	–	3	_	MW762644– MW762646
Mala#	Suvarna	13.2247	75.1369			NA	NA

*Type locality of *N. jog* as mentioned in Biju et al. (2011)*. #* Not sampled in this study.

**Table 2.** Kimura2 parameter pairwise genetic distance (%) among 36 *Nyctibatrachus* species from the Western Ghats based on 530 bp of mitochondrial 16S rRNA gene (1–23: GenBank Accession numbers of *N.jog*).

	**1**	**2**	**3**	**4**	**5**	**6**	**7**	**8**	**9**	**10**	**11**	**12**	**13**	**14**	**15**	**16**	**17**
																	
**2**	0																
**3**	0	0															
**4**	0	0	0														
**5**	0	0	0	0													
**6**	0	0	0	0	0												
**7**	0	0	0	0	0	0											
**8**	0	0	0	0	0	0	0										
**9**	0.84	0.84	0.84	0.84	0.84	0.84	0.84	0.84									
**10**	0.84	0.84	0.84	0.84	0.84	0.84	0.84	0.84	0								
**11**	0.84	0.84	0.84	0.84	0.84	0.84	0.84	0.84	0	0							
**12**	0.84	0.84	0.84	0.84	0.84	0.84	0.84	0.84	0	0	0						
**13**	0.63	0.63	0.63	0.63	0.63	0.63	0.63	0.63	0.21	0.21	0.21	0.21					
**14**	0.63	0.63	0.63	0.63	0.63	0.63	0.63	0.63	0.21	0.21	0.21	0.21	0				
**15**	0.63	0.63	0.63	0.63	0.63	0.63	0.63	0.63	0.21	0.21	0.21	0.21	0	0			
**16**	0.63	0.63	0.63	0.63	0.63	0.63	0.63	0.63	0.21	0.21	0.21	0.21	0	0	0		
**17**	0.63	0.63	0.63	0.63	0.63	0.63	0.63	0.63	0.21	0.21	0.21	0.21	0	0	0	0	
**18**	0.63	0.63	0.63	0.63	0.63	0.63	0.63	0.63	0.21	0.21	0.21	0.21	0	0	0	0	0
**19**	0.63	0.63	0.63	0.63	0.63	0.63	0.63	0.63	0.21	0.21	0.21	0.21	0	0	0	0	0
**20**	0.63	0.63	0.63	0.63	0.63	0.63	0.63	0.63	0.21	0.21	0.21	0.21	0	0	0	0	0
**21**	0.63	0.63	0.63	0.63	0.63	0.63	0.63	0.63	0.21	0.21	0.21	0.21	0	0	0	0	0
**22**	0.63	0.63	0.63	0.63	0.63	0.63	0.63	0.63	0.21	0.21	0.21	0.21	0	0	0	0	0
**23**	0.63	0.63	0.63	0.63	0.63	0.63	0.63	0.63	0.21	0.21	0.21	0.21	0	0	0	0	0
**24**	11.50	11.50	11.50	11.50	11.50	11.50	11.50	11.50	11.75	11.75	11.75	11.75	11.75	11.75	11.75	11.75	11.75
**25**	12.02	12.02	12.02	12.02	12.02	12.02	12.02	12.02	11.77	11.77	11.77	11.77	11.77	11.77	11.77	11.77	11.77
**26**	10.51	10.51	10.51	10.51	10.51	10.51	10.51	10.51	10.51	10.51	10.51	10.51	10.51	10.51	10.51	10.51	10.51
**27**	10.98	10.98	10.98	10.98	10.98	10.98	10.98	10.98	11.23	11.23	11.23	11.23	11.23	11.23	11.23	11.23	11.23
**28**	10.98	10.98	10.98	10.98	10.98	10.98	10.98	10.98	11.23	11.23	11.23	11.23	11.23	11.23	11.23	11.23	11.23
**29**	11.06	11.06	11.06	11.06	11.06	11.06	11.06	11.06	10.81	10.81	10.81	10.81	10.81	10.81	10.81	10.81	10.81
**30**	10.74	10.74	10.74	10.74	10.74	10.74	10.74	10.74	10.99	10.99	10.99	10.99	10.99	10.99	10.99	10.99	10.99
**31**	8.79	8.79	8.79	8.79	8.79	8.79	8.79	8.79	8.79	8.79	8.79	8.79	8.79	8.79	8.79	8.79	8.79
**32**	12.31	12.31	12.31	12.31	12.31	12.31	12.31	12.31	12.06	12.06	12.06	12.06	12.06	12.06	12.06	12.06	12.06
**33**	13.07	13.07	13.07	13.07	13.07	13.07	13.07	13.07	13.60	13.60	13.60	13.60	13.60	13.60	13.60	13.60	13.60
**34**	12.52	12.52	12.52	12.52	12.52	12.52	12.52	12.52	12.52	12.52	12.52	12.52	12.52	12.52	12.52	12.52	12.52
**35**	12.55	12.55	12.55	12.55	12.55	12.55	12.55	12.55	13.07	13.07	13.07	13.07	13.07	13.07	13.07	13.07	13.07
**36**	12.35	12.35	12.35	12.35	12.35	12.35	12.35	12.35	12.35	12.35	12.35	12.35	12.35	12.35	12.35	12.35	12.35
**37**	11.80	11.80	11.80	11.80	11.80	11.80	11.80	11.80	12.31	12.31	12.31	12.31	12.31	12.31	12.31	12.31	12.31
**38**	11.08	11.08	11.08	11.08	11.08	11.08	11.08	11.08	11.58	11.58	11.58	11.58	11.58	11.58	11.58	11.58	11.58
**39**	11.99	11.99	11.99	11.99	11.99	11.99	11.99	11.99	11.74	11.74	11.74	11.74	11.74	11.74	11.74	11.74	11.74
**40**	11.05	11.05	11.05	11.05	11.05	11.05	11.05	11.05	10.80	10.80	10.80	10.80	10.80	10.80	10.80	10.80	10.80
**41**	11.79	11.79	11.79	11.79	11.79	11.79	11.79	11.79	11.53	11.53	11.53	11.53	11.53	11.53	11.53	11.53	11.53
**42**	10.21	10.21	10.21	10.21	10.21	10.21	10.21	10.21	9.97	9.97	9.97	9.97	9.97	9.97	9.97	9.97	9.97
**43**	11.52	11.52	11.52	11.52	11.52	11.52	11.52	11.52	12.02	12.02	12.02	12.02	12.02	12.02	12.02	12.02	12.02
**44**	3.62	3.62	3.62	3.62	3.62	3.62	3.62	3.62	4.06	4.06	4.06	4.06	3.84	3.84	3.84	3.84	3.84
**45**	10.29	10.29	10.29	10.29	10.29	10.29	10.29	10.29	10.29	10.29	10.29	10.29	10.29	10.29	10.29	10.29	10.29
**46**	9.10	9.10	9.10	9.10	9.10	9.10	9.10	9.10	9.35	9.35	9.35	9.35	9.35	9.35	9.35	9.35	9.35
**47**	10.80	10.80	10.80	10.80	10.80	10.80	10.80	10.80	11.55	11.55	11.55	11.55	11.55	11.55	11.55	11.55	11.55
**48**	4.52	4.52	4.52	4.52	4.52	4.52	4.52	4.52	4.52	4.52	4.52	4.52	4.30	4.30	4.30	4.30	4.30
**49**	11.55	11.55	11.55	11.55	11.55	11.55	11.55	11.55	11.80	11.80	11.80	11.80	11.80	11.80	11.80	11.80	11.80
**50**	12.02	12.02	12.02	12.02	12.02	12.02	12.02	12.02	12.28	12.28	12.28	12.28	12.28	12.28	12.28	12.28	12.28
**51**	10.24	10.24	10.24	10.24	10.24	10.24	10.24	10.24	10.24	10.24	10.24	10.24	10.24	10.24	10.24	10.24	10.24
**52**	1.26	1.26	1.26	1.26	1.26	1.26	1.26	1.26	1.26	1.26	1.26	1.26	1.05	1.05	1.05	1.05	1.05
**53**	10.10	10.10	10.10	10.10	10.10	10.10	10.10	10.10	10.59	10.59	10.59	10.59	10.59	10.59	10.59	10.59	10.59
**54**	11.25	11.25	11.25	11.25	11.25	11.25	11.25	11.25	11.50	11.50	11.50	11.50	11.50	11.50	11.50	11.50	11.50
**55**	12.26	12.26	12.26	12.26	12.26	12.26	12.26	12.26	12.52	12.52	12.52	12.52	12.52	12.52	12.52	12.52	12.52
**56**	11.55	11.55	11.55	11.55	11.55	11.55	11.55	11.55	11.80	11.80	11.80	11.80	11.80	11.80	11.80	11.80	11.80
**57**	11.77	11.77	11.77	11.77	11.77	11.77	11.77	11.77	12.02	12.02	12.02	12.02	12.02	12.02	12.02	12.02	12.02
**58**	10.10	10.10	10.10	10.10	10.10	10.10	10.10	10.10	10.34	10.34	10.34	10.34	10.34	10.34	10.34	10.34	10.34

	**18**	**19**	**20**	**21**	**22**	**23**	**24**	**25**	**26**	**27**	**28**	**29**	**30**	**31**	**32**
**19**	0														
**20**	0	0													
**21**	0	0	0												
**22**	0	0	0	0											
**23**	0	0	0	0	0										
**24**	11.75	11.75	11.75	11.75	11.75	11.75									
**25**	11.77	11.77	11.77	11.77	11.77	11.77	7.44								
**26**	10.51	10.51	10.51	10.51	10.51	10.51	8.84	10.31							
**27**	11.23	11.23	11.23	11.23	11.23	11.23	8.81	12.30	8.64						
**28**	11.23	11.23	11.23	11.23	11.23	11.23	9.03	11.25	9.35	5.63					
**29**	10.81	10.81	10.81	10.81	10.81	10.81	8.46	8.44	8.67	11.08	9.54				
**30**	10.99	10.99	10.99	10.99	10.99	10.99	8.59	12.35	8.20	3.40	5.84	10.13			
**31**	8.79	8.79	8.79	8.79	8.79	8.79	9.04	11.77	7.94	5.63	4.27	9.33	4.94		
**32**	12.06	12.06	12.06	12.06	12.06	12.06	6.51	3.64	9.12	12.08	10.04	9.17	11.62	10.30	
**33**	13.60	13.60	13.60	13.60	13.60	13.60	7.44	9.85	8.49	9.16	9.62	10.39	8.69	9.42	9.63
**34**	12.52	12.52	12.52	12.52	12.52	12.52	10.72	9.55	10.86	13.62	11.05	10.05	13.64	11.57	9.33
**35**	13.07	13.07	13.07	13.07	13.07	13.07	6.97	9.36	8.25	8.67	9.13	10.39	7.96	8.93	8.66
**36**	12.35	12.35	12.35	12.35	12.35	12.35	9.53	11.55	9.17	10.34	9.09	11.60	10.61	9.37	11.58
**37**	12.31	12.31	12.31	12.31	12.31	12.31	8.59	11.08	8.69	8.63	9.12	11.86	8.16	8.89	9.62
**38**	11.58	11.58	11.58	11.58	11.58	11.58	7.65	9.62	7.51	8.18	8.18	9.63	7.24	8.43	8.43
**39**	11.74	11.74	11.74	11.74	11.74	11.74	3.19	6.99	9.31	9.52	10.22	8.69	10.27	10.50	6.05
**40**	10.80	10.80	10.80	10.80	10.80	10.80	7.46	8.43	10.07	11.53	11.48	6.10	9.83	10.52	8.67
**41**	11.53	11.53	11.53	11.53	11.53	11.53	7.20	3.86	9.35	12.57	9.54	8.67	10.59	9.80	2.33
**42**	9.97	9.97	9.97	9.97	9.97	9.97	10.25	12.30	8.86	3.61	6.74	10.58	3.62	4.48	12.04
**43**	12.02	12.02	12.02	12.02	12.02	12.02	8.80	11.52	8.18	7.94	7.21	10.07	7.71	6.79	9.81
**44**	3.84	3.84	3.84	3.84	3.84	3.84	10.08	10.31	10.30	10.77	10.99	10.86	11.28	9.05	10.84
**45**	10.29	10.29	10.29	10.29	10.29	10.29	8.62	9.60	2.97	8.19	8.16	8.94	7.49	7.01	10.13
**46**	9.35	9.35	9.35	9.35	9.35	9.35	7.46	8.43	8.87	11.31	10.75	4.52	10.11	8.84	9.16
**47**	11.55	11.55	11.55	11.55	11.55	11.55	7.92	9.86	10.31	11.03	11.23	5.41	10.33	10.03	10.61
**48**	4.30	4.30	4.30	4.30	4.30	4.30	10.52	11.00	9.30	8.81	9.99	8.86	9.29	9.04	11.53
**49**	11.80	11.80	11.80	11.80	11.80	11.80	6.08	8.19	9.12	10.10	10.04	7.06	9.65	9.83	8.43
**50**	12.28	12.28	12.28	12.28	12.28	12.28	4.52	7.93	10.07	10.02	10.73	9.21	10.05	11.24	6.99
**51**	10.24	10.24	10.24	10.24	10.24	10.24	10.03	9.53	5.18	10.05	8.82	8.62	9.36	8.63	8.83
**52**	1.05	1.05	1.05	1.05	1.05	1.05	11.50	11.77	10.26	11.48	10.00	10.07	11.24	9.03	12.06
**53**	10.59	10.59	10.59	10.59	10.59	10.59	10.03	9.12	8.22	9.37	8.64	10.61	9.63	9.39	8.90
**54**	11.50	11.50	11.50	11.50	11.50	11.50	4.51	8.15	9.81	9.76	9.27	8.46	8.83	9.29	7.44
**55**	12.52	12.52	12.52	12.52	12.52	12.52	4.29	8.15	10.77	10.25	9.99	9.90	10.29	10.75	7.68
**56**	11.80	11.80	11.80	11.80	11.80	11.80	7.70	9.86	9.58	11.28	10.73	4.73	10.08	9.78	9.86
**57**	12.02	12.02	12.02	12.02	12.02	12.02	3.85	6.99	10.53	10.26	10.00	9.17	9.56	10.52	6.75
**58**	10.34	10.34	10.34	10.34	10.34	10.34	7.24	9.17	9.37	10.83	11.02	4.76	10.13	9.09	8.93

	**33**	**34**	**35**	**36**	**37**	**38**	**39**	**40**	**41**	**42**	**43**	**44**	**45**	**46**	**47**	**48**	**49**	**50**
**34**	10.65																	
**35**	1.26	11.41																
**36**	8.96	12.12	8.47															
**37**	3.84	12.42	3.84	8.49														
**38**	3.85	10.67	2.97	8.73	2.97													
**39**	8.61	11.20	8.13	10.01	9.30	8.35												
**40**	11.10	12.26	10.84	11.58	10.55	11.08	7.69											
**41**	9.86	10.55	8.89	11.82	10.10	8.66	7.68	8.90										
**42**	10.83	14.06	10.08	11.06	9.58	9.82	11.23	10.78	11.27									
**43**	8.20	11.57	8.20	11.15	8.40	7.95	10.23	12.02	10.53	8.85								
**44**	11.84	13.02	11.84	11.60	11.53	10.81	10.56	11.13	9.83	10.72	10.07							
**45**	9.01	10.90	8.28	8.96	7.99	8.02	9.09	9.85	9.13	8.38	8.20	10.58						
**46**	10.13	10.53	9.88	10.61	11.08	10.11	8.16	3.62	8.90	10.56	11.30	9.39	9.14					
**47**	10.59	10.78	10.59	11.33	11.30	10.83	8.86	4.51	10.10	11.03	11.52	10.59	10.10	2.97				
**48**	11.80	13.02	10.80	12.08	12.52	10.53	11.00	10.56	10.52	9.26	10.53	5.42	9.32	8.15	10.31			
**49**	9.14	10.29	9.39	10.83	9.59	9.62	6.31	5.63	7.94	10.10	10.58	11.10	8.42	5.19	5.40	9.82		
**50**	8.63	10.73	8.63	11.02	10.27	9.56	5.19	8.67	8.63	10.75	10.01	10.59	10.34	7.74	8.89	10.53	7.03	
**51**	8.94	10.08	8.70	9.12	9.12	7.70	10.26	10.51	8.11	9.32	9.88	9.78	5.63	9.31	11.25	9.04	9.31	9.55
**52**	13.33	12.26	13.33	11.08	12.57	11.84	11.99	11.05	11.03	10.21	11.27	3.62	10.53	9.10	11.30	4.52	11.55	12.02
**53**	8.73	9.90	8.73	8.51	7.30	7.09	9.77	11.35	8.40	10.05	9.14	9.35	7.76	9.88	10.10	10.05	9.85	9.55
**54**	9.83	11.71	8.86	11.25	9.07	8.12	4.97	8.18	8.38	10.50	9.52	10.83	8.38	8.20	9.60	10.77	7.70	3.85
**55**	9.83	11.71	8.62	9.77	10.04	8.83	5.20	10.13	8.62	12.25	11.48	11.33	9.81	9.88	11.10	11.05	8.69	5.19
**56**	10.13	11.00	9.88	10.33	11.05	10.33	8.40	4.29	9.60	10.78	10.77	11.35	10.34	3.18	2.32	10.07	5.40	7.95
**57**	9.12	10.98	7.92	10.03	9.81	8.13	4.76	8.91	7.68	12.01	10.74	11.10	9.58	8.91	9.86	10.78	7.98	4.29
**58**	10.65	11.30	10.39	10.88	10.84	10.63	7.46	3.41	9.16	10.08	11.06	10.39	9.65	1.47	2.54	9.13	4.52	7.96

	**51**	**52**	**53**	**54**	**55**	**56**	**57**
**52**	9.52						
**53**	7.45	9.85					
**54**	8.82	11.75	9.78				
**55**	9.28	12.26	10.52	4.51			
**56**	9.31	11.05	10.34	8.42	9.88		
**57**	9.05	11.77	10.29	4.07	1.90	8.67	
**58**	10.05	10.10	10.39	8.43	9.90	2.75	9.17

1. MW081889, 2. KP317819, 3. JN644900, 4. MW081890, 5. MW081891, 6. MW081892, 7. MW081893, 8. MW081894, 9. MW081895, 10. MW081896, 11. MW081897, 12. MW081898, 13. MW081899, 14. MW081900, 15. MW081901, 16. MW081902, 17. MW081903, 18. MW081904, 19. MW081905, 20. MW081906, 21. MW081907, 22. MW081908, 23. MW081909, 24. *N. kumbara,* 25. *N. kempholeyensis,* 26.*N. webilla,* 27.*N. sabarimalai,* 28. *N. robinmoorei,* 29. *N. radcliffei,* 30. *N. pulivijayani,* 31.*N. manalari,* 32*. N.athirapillyensis,* 33. *N. periyar,* 34. *N. minimus* 35. *N. deveni*, 36. *N. vasanthi*, 37. *N. aliciae*, 38. *N. pillai*, 39. *N dattatreyaensis*, 40. *N. major*, 41. *N. mewasinghi*, 42. *N. anamallaiensis*, 43. *N beddomii*, 44. *N. danieli*, 45. *N. deccanensis*, 46. *N. gavi*, 47. *N. grandis*, 48. *N. humayuni*, 49. *N. indraneili*, 50. *N. karnatakaensis*, 51. *N. minor*, 52. *N. petraeus*, 53. *N. poocha*, 54. *N. sanctipalustris*, 55. *N. shiradi*, 56. *N. sylvaticus*, 57. *N. vrijeuni*, 58. *N. acanthodermis*.

**Table 3.** Kimura2 parameter pairwise genetic distance (%) between individuals of *Nyctibatrachus* jog from the Western Ghats based on 427 bp of mitochondrial Cyt-b gene.

	**1**	**2**	**3**	**4**	**5**	**6**	**7**	**8**	**9**	**10**	**11**	**12**	**13**	**14**	**15**
**2**	0														
**3**	0	0													
**4**	0	0	0												
**5**	0	0	0	0											
**6**	0	0	0	0	0										
**7**	0	0	0	0	0	0									
**8**	1.95	1.95	1.95	1.95	1.95	1.95	1.95								
**9**	1.95	1.95	1.95	1.95	1.95	1.95	1.95	0							
**10**	1.95	1.95	1.95	1.95	1.95	1.95	1.95	0	0						
**11**	1.95	1.95	1.95	1.95	1.95	1.95	1.95	0	0	0					
**12**	1.7	1.70	1.70	1.70	1.70	1.70	1.70	0.24	0.24	0.24	0.24				
**13**	1.7	1.70	1.70	1.70	1.70	1.70	1.70	0.24	0.24	0.24	0.24	0			
**14**	1.70	1.70	1.70	1.70	1.70	1.70	1.70	0.24	0.24	0.24	0.24	0	0	0	0
**15**	1.70	1.70	1.70	1.70	1.70	1.70	1.70	0.24	0.24	0.24	0.24	0	0	0	0
**16**	1.70	1.70	1.70	1.70	1.70	1.70	1.70	0.24	0.24	0.24	0.24	0	0	0	0
**17**	1.70	1.70	1.70	1.70	1.70	1.70	1.70	0.24	0.24	0.24	0.24	0	0	0	0
**18**	1.70	1.70	1.70	1.70	1.70	1.70	1.70	0.24	0.24	0.24	0.24	0	0	0	0
**19**	1.70	1.70	1.70	1.70	1.70	1.70	1.70	0.24	0.24	0.24	0.24	0	0	0	0
**20**	1.70	1.70	1.70	1.70	1.70	1.70	1.70	0.24	0.24	0.24	0.24	0	0	0	0
**21**	1.70	1.70	1.70	1.70	1.70	1.70	1.70	0.24	0.24	0.24	0.24	0	0	0	0
**22**	1.95	1.95	1.95	1.95	1.95	1.95	1.95	0.48	0.48	0.48	0.48	0.24	0.24	0.24	0.24
**23**	1.95	1.95	1.95	1.95	1.95	1.95	1.95	0.00	0	0	0	0.24	0.24	0.24	0.24
**24**	1.95	1.95	1.95	1.95	1.95	1.95	1.95	0.00	0	0	0	0.24	0.24	0.24	0.24
**25**	1.95	1.95	1.95	1.95	1.95	1.95	1.95	0.48	0.48	0.48	0.48	0.24	0.24	0.24	0.24
**26**	1.95	1.95	1.95	1.95	1.95	1.95	1.95	0.48	0.48	0.48	0.48	0.24	0.24	0.24	0.24

	**16**	**17**	**18**	**19**	**20**	**21**	**22**	**23**	**24**	**25**
**17**	0									
**18**	0	0								
**19**	0	0	0							
**20**	0	0	0	0						
**21**	0	0	0	0	0					
**22**	0.24	0.24	0.24	0.24	0.24	0.24				
**23**	0.24	0.24	0.24	0.24	0.24	0.24	0.48			
**24**	0.24	0.24	0.24	0.24	0.24	0.24	0.48	0.48		
**25**	0.24	0.24	0.24	0.24	0.24	0.24	0.48	0.48	0.48	
**26**	0.24	0.24	0.24	0.24	0.24	0.24	0.48	0.48	0.48	0.48

1. MW762640 2. MW762641, 3. MW762642, 4. MW762643, 5. MW762644, 6. MW762645, 7. MW762646, 8. MW762647, 9. MW762648, 10. MW762649, 11. MW762650, 12. MW762651, 13. MW762652, 14. MW762653, 15. MW762654, 16. MW762655, 17. MW762656, 18. MW762657, 19. MW762658, 20. MW762659, 21. MW762660, 22. MW762661, 23. MW762662, 24. MW762663, 25. MW762664, 26. MW762665.

## Discussion

The range extension of *N. jog* suggests either lack of fine-scale sampling by previous studies or the difficulty in sighting this species due to its specific microhabitat preferences. *N. jog* prefers fast flowing streams and waterfalls which may not be easily accessible for sampling leading to few observations. Even in this study, the sightings were only in 9 streams out of the 43 streams sampled. Seasonality could be another reason as *N. jog* calls are not heard post monsoon making it difficult to identify in the field. Also, *Nyctibatrachus* genus harbors cryptic species, hence previous studies based on morphological observations may have misidentified *N. jog* for another *Nyctibatrachus* species as it was earlier known to have restriction distribution. This DNA barcoding study now indicates the wide distribution of *N. jog* as against the previous notion that it is distributed only in the type locality of the Sharavathi river basin (Biju et al. 2011).

The habitats of *N. jog* are perennial fast-flowing streams and torrents. The major threats to its habitats are the conversion of the streams into areca plantations and paddy fields. Since *N. jog* inhabits torrents, there is a threat to their habitat by tourists who use the torrents for bathing and defecating. According to the International Union of Conservation of Nature (IUCN) Global Redlist assessment, the status of *N. jog* is not evaluated (Dandekar et al. 2020). From this study, it is evident that the EOO of *N. jog* has increased, thus it is no longer restricted to its type locality, Jog Falls of the Sharavathi river basin. Since this species appears to have wide distribution, efforts are needed for extensive fieldwork locating new populations. To increase the conservation efforts, multiple stakeholders must be involved that includes educating local people and forest department officials. Besides that, it is also essential to study population dynamics and population genetic studies for developing conservation strategies.

## Data Availability

The data that support the findings of this study are openly available in [NCBI] at https://www.ncbi.nlm.nih.gov/, reference numbers MW081889 to MW081909, MW762640 to MW762665. The voucher specimens mentioned in the study are deposited in Bombay Natural History Society (BNHS), Mumbai, email: rahul.bnhs@gmail.com.
